# Attenuation of the Diffuse Noxious Inhibitory Controls in Chronic Joint Inflammatory Pain Is Accompanied by Anxiodepressive-Like Behaviors and Impairment of the Descending Noradrenergic Modulation

**DOI:** 10.3390/ijms21082973

**Published:** 2020-04-23

**Authors:** Raquel Pereira-Silva, José Tiago Costa-Pereira, Raquel Alonso, Paula Serrão, Isabel Martins, Fani L. Neto

**Affiliations:** 1 Instituto de Investigação e Inovação em Saúde da Universidade do Porto (I3S). Rua Alfredo Allen 208, 4200-393 Porto, Portugal; raquel.silva.farm.86@gmail.com (R.P.-S.); jose.tiago.pereira@gmail.com (J.T.C.-P.); raquel_mna@hotmail.com (R.A.); isabmart@med.up.pt (I.M.); 2 Instituto de Biologia Molecular e Celular (IBMC), Universidade do Porto. Rua Alfredo Allen 208, 4200-393 Porto, Portugal; 3 Departamento de Biomedicina–Unidade de Biologia Experimental, Faculdade de Medicina, Universidade do Porto. Alameda Prof. Hernâni Monteiro, 4200-319 Porto, Portugal; 4 Departamento de Biomedicina–Unidade de Farmacologia e Terapêutica, Faculdade de Medicina, Universidade do Porto. Alameda Prof. Hernâni Monteiro, 4200-319 Porto, Portugal; mpvserrao@gmail.com; 5 MedInUP–Center for Drug Discovery and Innovative Medicines, University of Porto. Alameda Prof. Hernâni Monteiro, 4200-319 Porto, Portugal

**Keywords:** chronic joint inflammatory pain, descending noradrenergic pain modulation, diffuse noxious inhibitory controls, anxiodepressive comorbidities, locus coeruleus, pERK1/2

## Abstract

The noradrenergic system is paramount for controlling pain and emotions. We aimed at understanding the descending noradrenergic modulatory mechanisms in joint inflammatory pain and its correlation with the diffuse noxious inhibitory controls (DNICs) and with the onset of anxiodepressive behaviours. In the complete Freund’s adjuvant rat model of Monoarthritis, nociceptive behaviors, DNICs, and anxiodepressive-like behaviors were evaluated. Spinal alpha2-adrenergic receptors (a2-AR), dopamine beta-hydroxylase (DBH), and noradrenaline were quantified concomitantly with a2-AR pharmacologic studies. The phosphorylated extracellular signal–regulated kinases 1 and 2 (pERK1/2) were quantified in the Locus coeruleus (LC), amygdala, and anterior cingulate cortex (ACC). DNIC was attenuated at 42 days of monoarthritis while present on days 7 and 28. On day 42, in contrast to day 28, noradrenaline was reduced and DBH labelling was increased. Moreover, spinal a2-AR were potentiated and no changes in a2-AR levels were observed. Additionally, at 42 days, the activation of ERKs1/2 was increased in the LC, ACC, and basolateral amygdala. This was accompanied by anxiety- and depressive-like behaviors, while at 28 days, only anxiety-like behaviors were observed. The data suggest DNIC is attenuated in prolonged chronic joint inflammatory pain, and this is accompanied by impairment of the descending noradrenergic modulation and anxiodepressive-like behaviors.

## 1. Introduction

Joint inflammatory pain is a highly prevalent condition [1,2], and the available treatments are often inefficient and have various adverse side-effects [3]. The pathophysiological mechanisms underlying pain, in these chronic conditions, are still not completely understood [4,5,6,7,8].

Chronic pain involves many different complex processes in key spinal and supraspinal areas, which may suffer significant changes in an attempt to adapt to the ongoing noxious stimuli [4,5,7]. Indeed, the control of pain implies several molecular changes at the spinal cord, in the descending modulation of pain, and in supraspinal areas involved in the emotional component of pain processing [4,5,7,9,10,11]. The latter may be influenced and altered by the noxious stimuli and also may be responsible for changes in the way these pathologic stimuli are processed [9,12,13]. Despite this knowledge, much is still unclear about how and when these changes in pain processing occur during the progression of a chronic joint inflammatory painful condition. Indeed, the balance between facilitatory and inhibitory input is compromised in chronic pain [4,14]. Through the release of noradrenaline at the spinal cord, the descending noradrenergic system is implicated in the spinal inhibition of noxious input, modulating the transmission of nociceptive information [4,15]. This descending noradrenergic input is impaired in neuropathic pain conditions [16,17,18]. Although in joint inflammatory pain this is rather unexplored, the few studies performed suggest the presence of changes in this modulatory system [19,20]. These studies have mainly explored the diffuse noxious inhibitory controls (DNICs), which are α2 adrenoceptor (a2-AR)-mediated endogenous inhibitory mechanisms involved in descending pain modulation [19]. Normally, the DNIC are at play when the noxious activity of a set of neurons is highly inhibited by a simultaneous noxious stimulus that is spatially distant [21,22,23]. In the monoarthritis rat model induced by intraarticular injection of complete Freund’s adjuvant solution (CFA), Danziger et al. (1999) reported that the DNICs are present at acute stages, but it is lost at 3-4 weeks of disease evolution [20]. In a rat model of osteoarthritis at an early inflammatory stage, rats showed fully functioning DNIC, while at a later stage, the DNIC were lost [19]. However, studies suggest osteoarthritic pain has a strong neuropathic component at later stages [24,25,26], which is not observed in the monoarthritis inflammatory model, indicating that the underlying pathophysiologic mechanisms might be different. At early stages of osteoarthritis, spinal administration of an a2-AR selective antagonist completely eliminated DNIC [19], reinforcing that the DNIC are a reliable indicator of the functionality of the descending noradrenergic system [24]. Thus, while DNIC studies indicate that these circuits might be compromised, a thorough evaluation of the descending noradrenergic system is still needed in joint inflammatory pain.

The main spinal source of noradrenaline is the Locus coeruleus (LC), which presents a functional dichotomy, by modulating descending inhibition through its projections to the spinal cord, and by mediating emotional-related behaviors via its ascending targets [9,27]. Through these LC projections to brain regions implicated in the affective component of pain, such as the anterior cingulate cortex (ACC) and the amygdala, a noradrenergic impairment might be implicated on the onset of pain-induced emotional disorders [9,10,12]. Interestingly, Borges et al. have shown that, in the CFA monoarthritis model, the expression of the phosphorylated extracellular signal–regulated kinases 1 and 2 (pERKs1/2), a neuronal activation marker, was increased in the LC, as well as in ACC and amygdala at 28 days of MA, and this was concomitant with the onset of emotional behavioral comorbidities [9,12]. However, in chronic joint inflammatory pain conditions, it is unclear if these changes in supraspinal activity and affective behaviors correlate with alterations at the spinal level in the descending noradrenergic system.

In the present study, to understand how these spinal, descending modulation and pain-related affective circuits are affected by a chronic joint inflammatory pain condition, we have used the monoarthritis rat model to examine different components essential for the pain experience, namely the spinal nociceptive system, the descending pain modulatory system, and the affective component, with a special focus on the noradrenergic system. Hence, we have studied the noradrenergic modulatory system at the spinal cord level. Specifically, we have pharmacologically manipulated spinal a2-AR to assess its functional response and have evaluated spinal a2-AR levels. Additionally, we have evaluated noradrenaline levels and the immunolabeling of the noradrenaline’s biosynthetic enzyme, dopamine beta-hydroxylase (DBH), in the spinal cord. We have also assessed the DNIC as part of a strategy to study the integrity of the descending inhibitory noradrenergic system. Finally, to assess the integrity of the affective component in this model, we have evaluated pain-induced anxiety and depressive-like behaviors. We have further assessed the neuronal activity in the LC and in supraspinal affective-related areas associated with the pain’s emotional component by quantifying the immunolabeling of pERKs1/2, which is a marker of neuronal activity associated with these pain-induced anxiety and depressive-like behaviors in chronic joint pain models [9,10,28,29].

## 2. Results

### 2.1. Nociceptive Behavior Assessement in the Monoarthritis Pain Model: Nociceptive Hypersensitivity Is Maintained until Six Weeks of Monoarthritis

**Figure 1:**
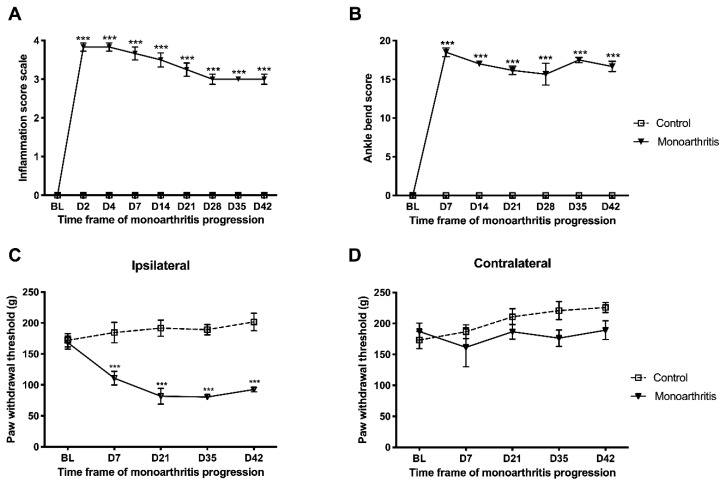
Evaluation of the development of inflammation and nociceptive behavior in control and monoarthritic rats. (**A**) Inflammation scores; (**B**) movement-induced allodynia, assessed by the ankle bend test; (**C**,**D**) paw withdrawal threshold observed in the Randall–Selitto test in the ipsilateral (**C**) and contralateral (**D**) paws. For the inflammation score and ankle bend test, only the ipsilateral paw values are shown in the graphs. For the contralateral paws, the values obtained for these parameters were null and, therefore, statistical tests could not be applied. Values expressed in Mean ± SEM. Six animals per group. *** *p* < 0.001. Two-way ANOVA with repeated-measures test followed by the Tukey’s post hoc test for multiple comparisons between the monoarthritic and control groups. BL = baseline; D = day.

The establishment and evolution of monoarthritis was evaluated before (i.e., baseline) its induction and during the 42-day experimental period via three different components: signs of inflammation and edema, assessed by the inflammatory score (Figure 1A and Table S1), movement-induced allodynia, assessed by the ankle bend test (Figure 1B and Table S1), and mechanical secondary hyperalgesia, assessed by the Randall–Selitto test (Figure 1C,D and Table S1).

After intraarticular injection, the monoarthritic rats developed intense edema and inflammation on the ipsilateral tibiotarsal joint, only a few hours after the procedure. This symptomatology increased on day 2 after CFA injection and remained stable for the 42 days of experimental period, as indicated by the high inflammatory scores (ipsilateral paw: *p* < 0.0001 for all significant time points; Figure 1A and Table S1). The control group, on the other hand, showed minor edema until day 7, mainly due to the mechanical damage provoked by the procedure, and the signs of inflammation ceased afterwards (Figure 1A and Table S1). Movement-induced allodynia was also observed on the ipsilateral ankle joints of the monoarthritic animals, but not of the control animals, from day 7 after CFA intra-articular injection until day 42, as shown by the high ankle bend scores (ipsilateral paw: *p* < 0.0001 for all significant time points; Figure 1B and Table S1). No signs of inflammation or movement-induced allodynia were significantly observed in the contralateral ankle joints.

The monoarthritic rats developed mechanical hyperalgesia in the ipsilateral hind paw, as shown by the decrease of mechanical thresholds on day 7, compared to baseline and to the ipsilateral hind paw of the control group. This decrease of the mechanical thresholds remained stable until day 42 (ipsilateral paw: *p* < 0.0001 for all significant timepoints; Figure 1C and Table S1). No differences were found between the control contralateral and the monoarthritic contralateral hind paws throughout the testing period (contralateral paw—baseline: *p* = 0.9800, day 7: *p* = 0.7872, day 21: *p* = 0.8116, day 28: *p* =0.2306, day 42: *p* =0.4305; Figure 1D and Table S1). None of the animals showed major abnormal variations on weight gain or loss during the 42 days of experimental period.

### 2.2. Characterization of the Spinal Noradrenergic Nociceptive System in Monoarthritis

#### 2.2.1. Monoarthritis Potentiates Spinal a2-AR Function without Changes in the Expression of Spinal a2-AR

We studied the effects of monoarthritis on spinal a2-AR at a prolonged time of disease, i.e., at 42 days, by evaluating the effects of the activation of a2-AR on mechanical hyperalgesia through the intrathecal injection of the selective agonist clonidine. In addition, the levels of a2A-AR subtype were quantified in the spinal dorsal horn.

**Figure 2:**
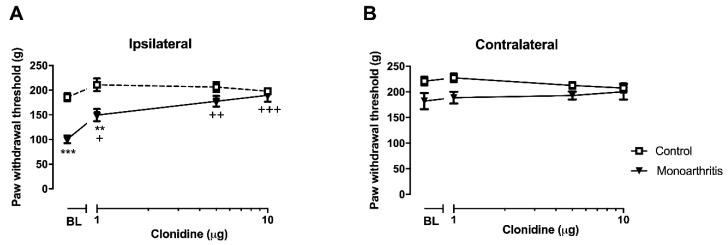
Effects of intrathecal cumulative doses of clonidine on the nociceptive responses in the ipsilateral (**A**) and contralateral paws (**B**) of control and monoarthritic rats. (**A**) The cumulative doses of clonidine induced a significant increase of the mechanical withdrawal threshold of the ipsilateral paw in monoarthritic rats, but not in control rats. (**B**) In the contralateral paw, no significant differences were found between the monoarthritis and control groups. Values expressed in Mean ± SEM. Six animals per group. Two-way ANOVA with repeated-measures test followed by Tukey’s multiple comparisons post hoc test. ** *p* < 0.01; *** *p* < 0.001, for comparison between the monoarthritis and control group; ^+^ *p* < 0.05; ^++^ *p* < 0.01; ^+++^ *p* < 0.001, for comparisons between each clonidine dose and the baseline before the first clonidine intrathecal injection. BL= baseline before the first clonidine intrathecal injection.

In the pharmacological studies, the analysis of the effects of cumulative doses of clonidine on the withdrawal thresholds of the ipsilateral paw in the control group showed that clonidine produced no significant effects compared to the baseline (values obtained before the first injection of clonidine; *p* = 0.3362, *p* = 0.5130, and *p* = 0.8435, respectively for doses 1 µg, 5 µg, and 10 µg; Figure 2A and Table S1). In contrast, in the monoarthritic rats, all doses of clonidine significantly increased the withdrawal thresholds compared to before the first injection of clonidine (*p* = 0.0344, *p* = 0.0001, and *p* < 0.0001, respectively for doses 1 µg, 5 µg, and 10 µg; Figure 2A and Table S1). At 5 µg and 10 µg doses, clonidine increased the withdrawal threshold to values similar to those of control animals (*p* = 0.2104 and *p* = 0.9670, respectively; Figure 2A and Table S1), which indicates a full reversion of mechanical hyperalgesia. Control and monoarthritic animals were only significantly different at baseline and after injection of the lower dose of clonidine (1 µg; *p* < 0.0001 and *p* = 0.0016, respectively; Figure 2A and Table S1). Clonidine produced no effects in the withdrawal thresholds of the contralateral paws of control and monoarthritis animals (Figure 2B and Table S1).

A supplementary group of control and monoarthritic animals were intrathecally injected with saline, and its effects were also evaluated. No differences in the paw withdrawal thresholds of the ipsi- or contralateral paws were found when compared with the baseline (Table S2). Therefore, these saline groups were not included in the analysis.

**Figure 3:**
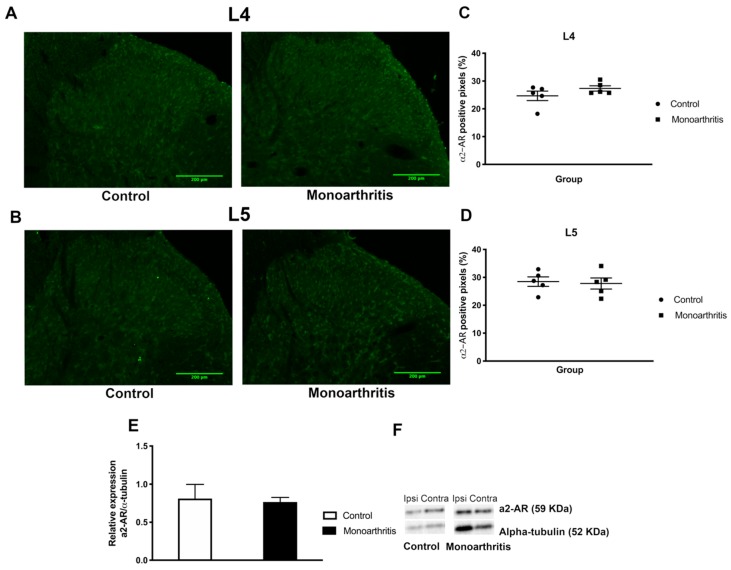
Effects of chronic inflammatory joint pain on a2A-AR protein levels in the spinal cord of control and monoarthritic rats quantified by immunofluorescence and western blot. Immunofluorescence labelling for a2A-AR in the spinal cord dorsal horn (laminae I-VI) of L4 (**A**) and L5 (**B**) segments in control (left) and monoarthritic (right) rats. (**C**,**D**) Percentage of a2A-AR-positive pixels in spinal cord segments L4 (**C**) and L5 (**D**). No significant changes were detected between monoarthritic and control animals. (**E**) Relative expression of a2A-AR in the spinal segments L4 and L5, normalized against alpha-tubulin levels. No significant difference was found in a2A-AR levels between monoarthritic and control groups. (**F**) Representative blot showing a2-AR (59 kDA) and alpha-tubulin (52 kDA, loading control) bands for the ipsilateral and contralateral sides of the spinal cord of a control (left) and a monoarthritic (right) animal. Values expressed in Mean ± SEM. Unpaired t-test for comparisons between the monoarthritic and control group in the immunofluorescence; five animals per group. Mann–Whitney non-parametric test for comparisons between the monoarthritic and control group in the western blot; four animals per group.

The a2A-ARs were quantified on the entire spinal dorsal horn by densitometric analysis of the western blot bands and of the immunofluorescence labelling. No significant changes were found in the percentage of a2A-AR positive pixels in the dorsal horn of monoarthritic animals in comparison with the control group, neither in the L4 nor in L5 spinal segments (*p* = 0.2092 and *p* = 0.8025 for L4 and L5 respectively; Figure 3A–D and Table S3), indicating that the spinal immunolabelling of a2A-AR was not affected by the joint inflammatory pain condition. These data were confirmed by western blot analysis of the dorsal horn of the spinal cord tissue. Quantification of a2A-AR band showed no statistically significant differences between the monoarthritic and control groups (*p* = 0.5000; Figure 3E–F and Table S3), supporting the immunohistochemical results. Because the distribution of the a2A-AR is mostly located in spinal dorsal horn laminae I and II, we also performed the densitometric analysis of the immunofluorescence labelling in these superficial layers to exclude any dilution of the immunohistochemical signal. This analysis showed no significant changes in the percentage of a2A-AR positive pixels of monoarthritic animals in comparison with the control group, neither in the L4 nor in L5 spinal segments (*p* = 0.6454 and *p* = 0.3987 for L4 and L5, respectively; Figure S1 and Table S3).

#### 2.2.2. Monoarthritis Alters the Noradrenaline Input to the Spinal Cord

**Figure 4:**
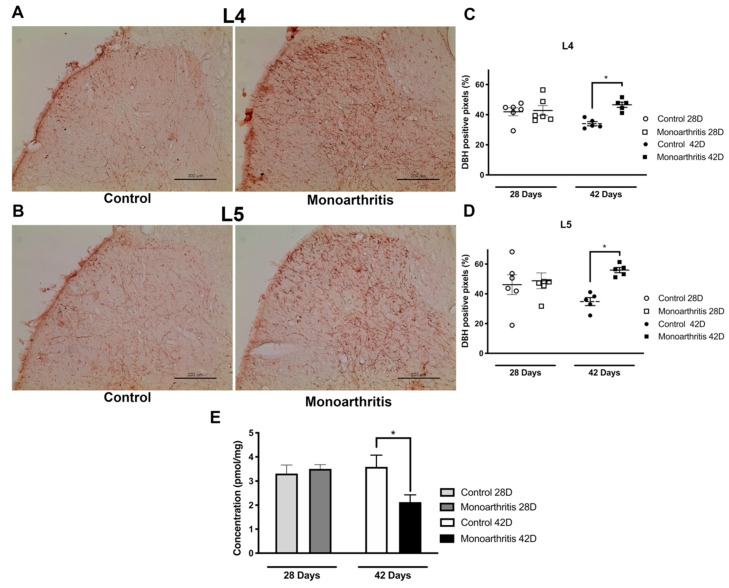
Effects of chronic inflammatory joint pain on the DBH labelling and noradrenaline concentration in the spinal cord of control and monoarthritic rats at 28 and 42 days after intraarticular injection, quantified by densitometric analysis and high-performance liquid chromatography (HPLC), respectively. (**A**,**B**) At 42 days, the monoarthritic rats (right) show significantly more DBH immunolabelled fibers in superficial laminae of L4 (A) and L5 (**B**) spinal cord segments than controls (left), but the same is not observed at 28 days. (**C**,**D**) Monoarthritis induced a significant increase in percentage of DBH positive pixels in spinal cord segments L4 (**C**) and L5 (**D**) at 42 days after intraarticular injection, as compared to controls (*n* = 5, for both experimental groups). No significant differences were found between groups at 28 days (*n* = 6, for both experimental groups); (**E**) At 42 days of monoarthritis, but not at 28 days, significant differences were found in the levels of noradrenaline on spinal segments L4 and L5 (pmol/mg) between control and monoarthritic animals groups, pointing to a decrease in the concentration of this neurotransmitter in the monoarthritic rats in comparison to the non-diseased group (*n* = 10 for controls and *n* = 9 for monoarthritis at 42 days; *n* = 6 for both experimental groups at 28 days). Values expressed in Mean ± SEM. * *p* < 0.05; One-way ANOVA followed by the Tukey’s post hoc test for multiple comparisons between groups.

In order to assess whether at 42 days of disease, monoarthritis induces neurochemical changes on the spinal noradrenergic system, we evaluated the basal levels of noradrenaline and the labeling for DBH, the noradrenaline rate-limiting enzyme, in the spinal dorsal horn of monoarthritic and control animals. The quantification of noradrenaline levels was performed separately in the ipsilateral and contralateral sides of the spinal cord dorsal horn of control and monoarthritic rats by high-performance liquid chromatography (HPLC). The overall concentration of noradrenaline was significantly lower in the monoarthritic rats compared to the control group (*p* = 0.0315; Figure 4E and Table S3). Immunohistochemistry was used to evaluate DBH labelling. A significant increase in the immunolabelling for DBH fibers was found bilaterally in the spinal dorsal horn of monoarthritic rats in comparison to control rats. This increase in DBH labelling was similar in both L4 and L5 spinal segments (*p* = 0.0174 and *p* = 0.0425 for L4 and L5, respectively; Figure 4A,B and Table S3). The findings observed at the microscopic level were translated into a statistically significant increase in the percentage of DBH positive labelling in spinal cord segments L4 and L5 (Figure 4C,D and Table S1) of monoarthritic animals, as determined by densitometric quantification.

We also determined whether the noradrenaline levels and DBH labelling were altered at 28 days of disease. At this earlier time point, no differences in noradrenaline levels (*p* = 0.9890; Figure 4E and Table S3) nor in DBH labelling were found (*p* = 0.9948 and *p* = 0.9786 for L4 and L5, respectively; Figure 4C,D and Table S3) between groups.

### 2.3. Characterization of the Descending Pain Modulatory System in Monoarthitis: The DNIC Is Present in Monoarthritic Animals, but Its Effects Are Attenuated at Six Weeks of Monoarthritis

DNIC tests were performed at days 7, 28, and 42 after CFA or CFA-vehicle intraarticular injection. As observed in the other monoarthritic animals of the study, monoarthritis was well developed and maintained throughout the 42-day experimental period in the group injected with CFA (data not shown), in contrast to the control group.

**Figure 5:**
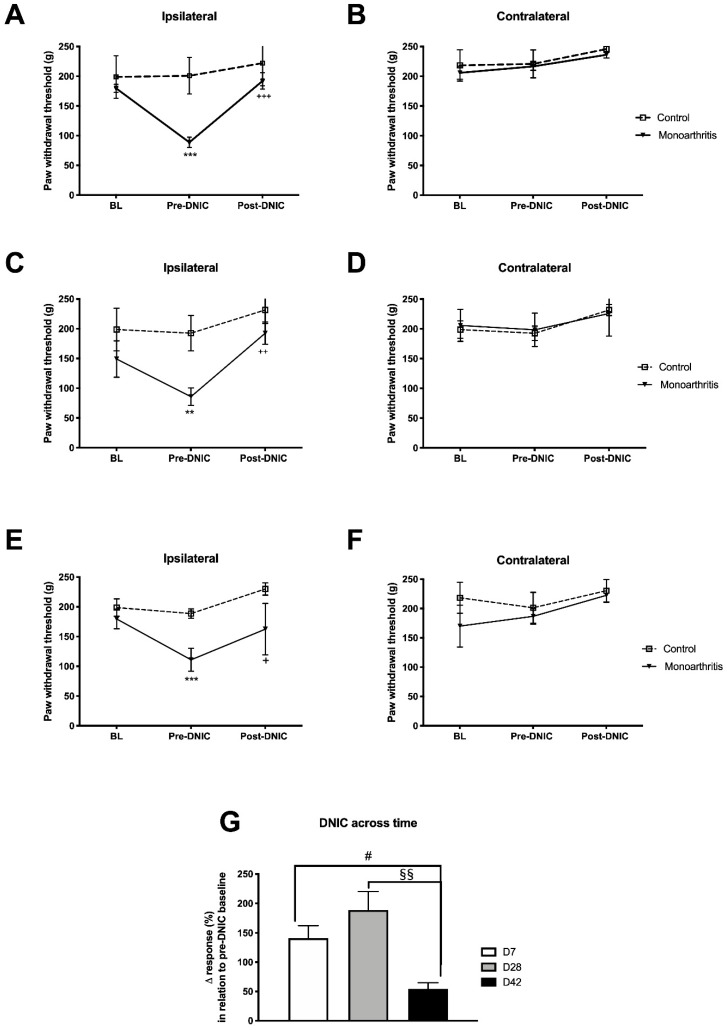
Effects of the diffuse noxious inhibitory control (DNIC) stimulation on the nociceptive responses of the ipsilateral and contralateral paws of control and monoarthritic rats on day 7 (**A**,**B**), 28 (**C**,**D**), and 42 (**E**,**F**) post intraarticular injection of complete Freund’s adjuvant solution (CFA)-vehicle or CFA. For all time points, the values of force needed for paw withdrawal (g) before intraarticular injection (BL) and at pre- and post-DNIC stimulation are presented. Post-DNIC stimulation significantly increased the withdrawal threshold on the ipsilateral hind paw of monoarthritic rats at all time points, in comparison to pre-DNIC and to the same paw in the control group. The DNIC magnitude was significantly reduced on day 42 compared to days 7 and 28 (**G**). Values expressed in Mean ± SEM. Six rats per group. Two-way ANOVA with repeated-measures test followed by Tukey’s multiple comparisons post hoc test; ** *p* < 0.01; *** *p* < 0.001, for comparisons between the monoarthritic and control group. ^+^ *p* < 0.05; ^++^ *p* < 0.01; ^+++^ *p* < 0.001, for comparisons between pre- and post-DNIC withdrawal thresholds in the monoarthritic rats. One-way ANOVA followed by Tukey’s multiple comparisons post hoc test; # *p* < 0.05; §§ *p* < 0.01, for comparisons between days 7, 28, and 42. BL = baseline before intraarticular injection; D = day.

Before the DNIC stimulus, the withdrawal thresholds of the ipsilateral paw of monoarthritic animals, but not of control animals, were significantly lower when compared to the baseline (i.e., before monoarthritis induction; day 7: *p* < 0.0001, day 28: *p* = 0.0482, day 42: *p* = 0.0006; Figure 5A–F and Table S1) and when compared to the control group at the three experimental time points tested (day 7: *p* < 0.0001, day 28: *p* = 0.0007, day 42: *p* = 0.0021; Figure 5A–F and Table S1), which shows the development of mechanical hyperalgesia following CFA injection. After DNIC, the withdrawal thresholds of the ipsilateral paw of monoarthritic rats were significantly increased, as compared to pre-DNIC, to values similar to or above those of the baseline, at the three experimental time points (pre-DNIC versus post-DNIC—day 7: *p* < 0.0001, day 28: *p* = 0.0072, day 42: *p* = 0.0498; baseline versus post-DNIC—day 7: *p* = 0.7590, day 28: *p* = 0.2225, day 42: *p* = 0.5552; Figure 5A–F and Table S1). DNIC produced no effects on the ipsilateral paws of control animals (Figure 5A–F; Supplementary Table S1). The analysis of the effects of DNIC in the contralateral paws of monoarthritic and control animals showed no significant differences, as no significant effects were induced by DNIC at the three time points tested (Figure 5A–F and Table S1).

We also evaluated the magnitude of the DNIC analgesia at each time point by determining the percentage of change induced by DNIC (Figure 5G and Table S3). This analysis showed that on day 42, DNIC induced a significantly lower percentage of variation compared to days 7 and 28 (day 7 versus day 42: *p* = 0.0392, day 28 versus day 42 = 0.0073; Figure 5G and Table S3), which indicates an attenuation of DNIC at this prolonged time point.

### 2.4. Characterization of the Affective Component of Pain Dimension in Monoarthritis

#### 2.4.1. Animals Show Anxiety and Depressive-Like Behaviors at Six Weeks of Monoarthritis

To determine the onset of anxiety- and/or depressive-like behaviors in chronic inflammatory joint pain, two tests were performed at 28 and 42 days after intraarticular injection.

**Figure 6:**
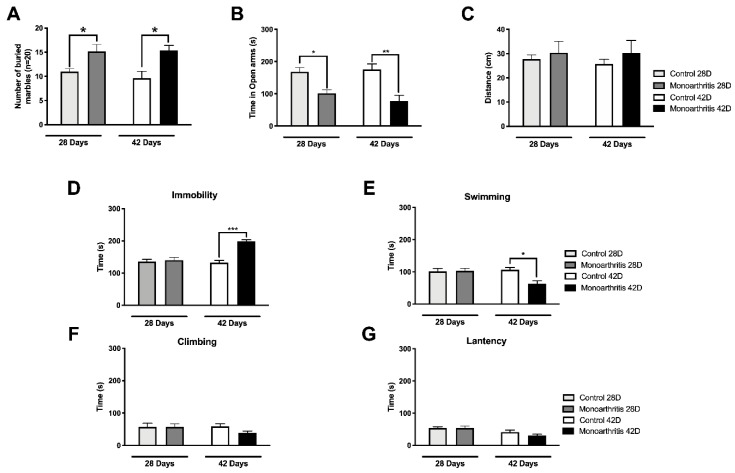
Anxiodepressive-like behaviors in monoarthritic rats at 28 and 42 days post intraarticular CFA injection. (**A**) MB: The monoarthritic group hid more marbles than the control group, showing anxiety-like behavior at both time points; (**B**) EZM: At 28 and 42 days of monoarthritis, monoarthritic rats spent less time in the open arms in comparison to the control group, which is another indication of anxiety-like behavior. (**C**) No differences in the distance travelled in the EZM test were found between groups. (**D**–**G**) FST: At 42 days, the monoarthritic rats spent more time immobile (**D**) and less time swimming (**E**) in comparison to the control group, therefore showing pain-induced depressive-like behaviors. No significant differences were found on climbing (**F**) and latency to immobility (**G**) behaviors. At 28 days, no significant differences were found between groups in any of the analyzed parameters. Values expressed in Mean ± SEM. Six and five animals per group at 28 and 42 days of monoarthritis, respectively. One-way ANOVA followed by the Tukey’s post hoc test for multiple comparisons between groups; * *p* < 0.05; ** *p* < 0.01; *** *p* < 0.001 for comparisons between the monoarthritic and control groups.

At 28 days, the monoarthritic rats hid a higher number of marbles than the control group in the marble-burying test (MB) (*p* = 0.0182; Figure 6A and Table S3) and spent significantly less time in the open arms of the elevated-zero maze (EZM) labyrinth in comparison to the control group (*p* = 0.0171; Figure 6B and Table S3), which are both an indication of anxiety-like behavior. At this time point, no significant differences were found between groups in the latency to immobility (*p* > 0.9999) and the time spent immobile (*p* = 0.9550), swimming (*p* = 0.9996) or climbing (*p* > 0.9999) evaluated in the forced swimming test (FST) (Figure 6D–G and Table S3). This suggests that depressive-like behaviors were not present at 28 days of monoarthritis.

At 42 days, a higher number of marbles were hidden by the monoarthritic animals (*p* = 0.0194; Figure 6A and Table S3), and significantly less time was spent in the open arms of the EZM, in comparison to the control group (*p* = 0.0015; Figure 6B and Table S3), an indication of anxiety-like behavior. Additionally, the monoarthritic rats demonstrated the development of depressive-like behavior since they spent more time immobile than the control group (*p* < 0.0001; Figure 6D and Table S3) and less time swimming (*p* = 0.0144; Figure 6E and Table S3). No differences were found between groups in the time spent climbing (*p* = 0.4847; Figure 6F and Table S3) or in latency to immobility (*p* = 0.6186; Figure 6G and Table S3).

At both time points, no significant differences were found in the distance travelled by monoarthritic or control groups in the EZM labyrinth (day 28: *p* = 0.9539, day 42: *p* = 0.8418; Figure 6C and Table S3), indicating no significant changes in the locomotor activity of the monoarthritic rats despite the nociceptive behavior and the high level of inflammation exhibited.

#### 2.4.2. Monoarthritis Increases Neuronal Activity in Supraspinal Affective-Related Areas Associated with the Emotional Component of Pain

The activation of ERK1/2 was analyzed in brain sections of monoarthritic and control animals by quantifying either the immunolabeling to pERKs1/2 in the LC or the number of pERKs1/2-immunoreactive (IR) cells in the entire rostrocaudal extension of the ACC and amygdala (basolateral nucleus of the amygdala (BLa) and medial amygdala (Me)). The ACC and amygdala are supraspinal areas that receive noradrenaline input from the LC and are implicated on the emotional component of pain. This study was performed at 42 days of the disease, when both anxiety and depression-like behaviors were observed.

**Figure 7:**
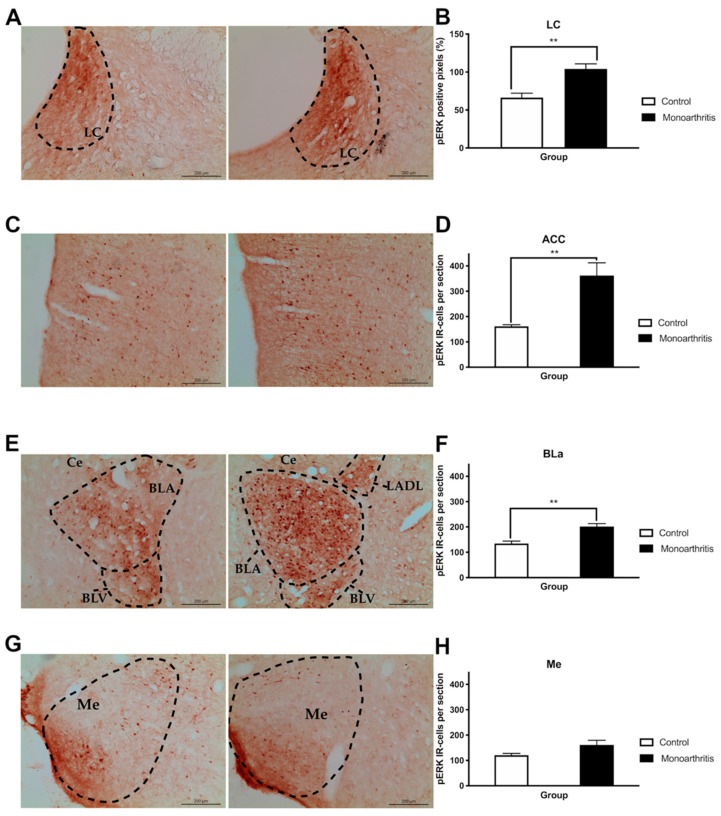
Activation of ERK1/2 in the Locus coeruleus (LC), anterior cingulate cortex (ACC), basolateral amygdala (BLa), and medial amygdala (Me) of monoarthritic and control rats at 42-days post-intraarticular injection was assessed by quantification of the immunolabeling for its phosphorylated form. (**A**,**C**,**E**,**G**) Immunoreaction for pERK1/2 in the LC (**A**), ACC (**C**), BLa (**E**), and Me (**G**) of control (left) and monoarthritic (right) rats. (**B**) Monoarthritis induced a significant increase in percentage of pERK1/2 positive pixels in the LC. (**D**,**F**,**H**) The monoarthritic rats show a higher number of pERK1/2-IR cells in the ACC (**D**) and BLa (F) but not in the Me (**H**); Values expressed in Mean ± SEM. 5 animals per group. ** *p* < 0.01; Unpaired t-test for comparisons between the monoarthritic and control groups. The black dashed lines are delineating the areas of interest. ACC = anterior cingulate cortex; BLA = Basolateral amygdala (anterior part); BLV = basolateral amygdala (ventral part); Ce = central amygdala; LaDL = lateral amygdaloid nucleus (dorsolateral part); LC = Locus coeruleus; Me = medial amygdala.

In the LC, the percentage of pERK1/2 positive pixels was higher in the monoarthritic group than in the control animals (*p* = 0.0025; Figure 7A,B and Table S3). In the ACC, the monoarthritic rats showed an increased number of pERK-IR cells in comparison to the control group (*p* = 0.0039; Figure 7C,D and Table S3). A similar significant increase of pERK IR-cells was also verified in the BLa of monoarthritic animals (*p* = 0.0018; Figure 7E,F and Table S3). In contrast, this pattern was not observed in the Me, as no significant changes in the number of pERK-IR cells were found between the two experimental groups (*p* = 0.0691; Figure 7G,H and Table S3).

## 3. Discussion

This is the first study, to the best of our knowledge, to show a stable pattern of increased nociception in the monoarthritic rat model of chronic inflammatory joint pain during the course of 42 days associated with an attenuation of DNIC at this prolonged time point of disease. Additionally, this is accompanied by anxiety- and depressive-like behaviors, while at 28 days after the CFA intraarticular injections, only anxiety-like behaviors are observed. Our study also showed that at 42 days of monoarthritis, there is a decrease in spinal noradrenaline content despite the augmented levels of DBH, but the same is not observed at 28 days. At the 42-day time point, these changes were also accompanied by a potentiation of spinal a2-AR without concomitant changes in the levels of this receptor. At the supraspinal level, increased activation of ERKs1/2, considered as a marker of neuronal activity associated with pain-induced anxiodepressive-like behaviors [9,12], was observed in the LC—the main source of noradrenaline input to the spinal cord—and to cortical and subcortical regions implicated in the control of the affective pain dimension, such as the ACC and amygdala. Interestingly, increased pERKs1/2 labeling was also found in those areas. Altogether, these findings suggest alterations in the noradrenergic control of nociception in the spinal cord at prolonged joint inflammatory pain conditions, which are accompanied by emotion-related pain-induced behaviors.

The monoarthritic rats manifested the expected symptomatology [9,12] having developed intense edema and inflammation and movement-induced allodynia in the ipsilateral hind paw accompanied by the development of mechanical hyperalgesia with an early onset, which was stably maintained throughout the six-week experimental period. With the exception of Millecamps et al. (2005), who evaluated nociception in the CFA monoarthritis model until 42 days [30], the common duration for the experiments in this model is not extended beyond 28 days [9,12]. Contrary to our study, Millecamps et al. (2005) showed that the mechanical hyperalgesia evaluated by the paw pressure test increased progressively until day 28, declining thereafter [30].

The main goals of our study were to examine and characterize the different components essential for the pain experience in the monoarthritic rat model of chronic inflammatory joint pain with particular emphasis on the noradrenergic system. We used the assessment of the DNIC as a strategy to study the integrity of the descending inhibitory noradrenergic system at different time points of disease. In the monoarthritic rats, the DNIC were present ipsilaterally at the three time points assessed, namely at early (seven days), intermediate (28 days), and prolonged (42 days) time points. This corroborates other studies indicating that DNIC events are active in chronic pain states [20,23,31,32,33]. DNIC were previously found in the carrageenan model of acute monoarthritis [31], in the CFA-induced monoarthritis model [20], and in the monoiodoacetate-induced osteoarthritis [19], a model of joint pain with a neuropathic component at the later stages [24,25,34]. Danziger et al. (1999) showed that the DNIC are present at acute stages (24–48 h) and are completely abolished at later stages (3–4 weeks) of CFA-induced monoarthritis [20]. To the best of our knowledge, this was the only study assessing the DNIC in the CFA-induced monoarthritis model of chronic joint inflammatory pain. In contrast to Danziger et al. (1999) studies [20], we show for the first time that, in the same joint pain model, that DNIC are present throughout the six weeks of disease development. The divergence in the results might be due to the application not only of different DNICs inducing stimuli but also in different body areas (contralateral paw vs. the tail in our study) of the diseased animals. In the present study, although the DNIC are still active at 42 days of monoarthritis, it is very much attenuated compared to the earlier time points evaluated. An attenuation of the DNIC at later disease stages was previously described by Lockwood et al. (2019) in the monoiodoacetate-induced osteoarthritis [19] and, also in a model of neuropathic pain, where the DNIC were absent [19]. In the early stage of monoiodoacetate-induced osteoarthritis, the spinal administration of a a2-AR selective antagonist completely abolished the DNIC. In addition, in the monoiodoacetate-induced osteoarthritis model at a later stage and in the neuropathic pain model, the DNIC were reestablished by the administration of a noradrenaline reuptake inhibitor (NRI) [19,23]. These findings indicate that the DNIC are a unique form of endogenous inhibitory control mediated majorly by the noradrenergic system, via a2-AR [19,23]. Therefore, the attenuation of the DNIC at 42 days of CFA-induced monoarthritis is likely related with an impairment of the descending noradrenergic modulatory system.

In this study, we also characterized the spinal noradrenergic nociceptive system at two different time points of the chronic stages of monoarthritis. Indeed, in agreement with our hypothesis of an impairment of the descending noradrenergic modulatory system at 42 days of CFA-induced monoarthritis, we found direct evidence of downregulation in the descending inhibitory noradrenergic modulation of the spinal cord. Indeed, on day 42 of monoarthritis but not at earlier time points (28 days), the monoarthritic rats showed a significant reduction in the spinal noradrenaline concentration, with a consequent diminished descending inhibitory input. This likely indicates an impairment of the spinal noradrenaline metabolism, probably due to an increased recruitment of noradrenaline inhibition in response to the ongoing nociceptive input. At 42 days, the downregulation of noradrenaline might reflect the progressive exhaustion of the noradrenaline descending inhibition, which is supported by the attenuation of the DNIC at this time point. The decreased levels of spinal noradrenaline could have two different causes: (1) the noradrenaline transporters (NET), which are responsible for the reuptake of extracellular noradrenaline, might be malfunctioning or overexpressed, causing an abnormal noradrenaline reuptake and leaving low concentrations of this neurotransmitter in the synaptic cleft [16,35]; and/or (2) monoamine oxidase (MAO), the enzyme involved in the degradation of noradrenaline, might be overexpressed or hyperactivated, increasing the degradation of noradrenaline [16].

In the monoarthritic animals, a potentiation of spinal a2-AR function without concomitant changes in the a2-AR levels was also found at this time point. The lack of effects of clonidine in the control animals is likely due to the fact that the descending noradrenergic pathways have a low activity in the absence of ongoing noxious input, as previously shown by the administration of a2-AR antagonists [36,37]. The potentiation of a2-AR in the monoarthritic animals reflects a compensatory activation of intracellular signaling cascades mediated by the receptor which leads to a hypersensitization state of the a2-AR. This was also observed by Bantel et al. (2005) in a neuropathic pain model, where an increased maximal functional binding efficacy of a2-AR coupled G-proteins was observed at the spinal cord dorsal horn [38]. It is likely that the effect observed in the a2-AR pharmacologic studies, where cumulative increasing doses of clonidine induce an increased antinociceptive effect in the ipsilateral hind paw of the monoarthritic animals, is due to the downregulation of noradrenaline we have also detected. Indeed, to the best of our knowledge, we are for the first time reporting a decrease in spinal noradrenaline associated with an a2-AR hypersensitization without changes in the levels of these receptors at prolonged chronic stages of monoarthritis. This decrease in noradrenaline in pre-synaptic neurons might be causing the post-synaptic neurons to compensate the lack of noradrenergic input by activating the intracellular signaling cascades mediated by the a2-AR, which leads to a hypersensitization state of these receptors.

The monoarthritic rats also showed increased DBH labelling at 42 days of monoarthritis, which was not observed on day 28. This further corroborates that the noradrenergic system is compensating the increased need of spinal noradrenaline by a higher recruitment of the biosynthetic machinery. It is likely that the low noradrenaline concentration might be messaging presynaptic neurons to increase the DBH production in the local axonal terminals in an attempt to counteract the low noradrenaline levels. In our joint inflammatory pain model, no significant changes were detected at 28 days, but an increased recruitment of the descending noradrenergic system was proposed to occur at early stages of development in neuropathic pain models [39,40], which was accompanied by increased spinal DBH [11,39].

Finally, to assess the integrity of the affective component in the monoarthritis model, we evaluated pain-induced anxiety and depressive-like behaviors on days 28 and 42 after the intraarticular injections and we quantified the pERK1/2 activity at the LC, ACC, and amygdala on day 42. Interestingly, the DNIC attenuation at 42 days after the indution of monoarthritis with CFA was also concomitant with changes in the neuronal activity of several cortical and subcortical brain areas, which were evaluated by the activation of ERK1/2. The ERK1/2 are enzymes that have been used for a few years as markers of neuronal activity associated with painful conditions and, with the development of pain-induced emotional comorbidities, such as anxiety- and depressive-like behaviors [12,28]. At 42 days, we found that the activation of ERK1/2 was highly increased in the LC of monoarthritic rats, indicating a rise in neuronal activity [28]. This increased neuronal activity is in many ways triggered by a response of the LC to the ongoing chronic noxious stimulation. However, since the LC is the main producer of noradrenaline in the central nervous system, it is possible that this increase in neuronal activity might be partly associated with an attempt to compensate for the noradrenergic impairment in the monoarthritis rat model. Thus, this increased activity could be correlated with an increased production of spinal DBH, as was detected, in an attempt to compensate the reduction of the noradrenaline content found in the spinal cord. In addition to the projections to the spinal cord, the LC is also connected to supraspinal regions that are involved in the control of the affective pain dimension and the development of pain-induced emotional comorbidities [27,41,42], such as the ACC and BLa [9,12,43,44,45,46]. Increased pERK1/2 labelling was also found in these brain areas in the monoarthritic rats. In contrast, no changes were detected in the Me, a nucleus of the amygdala that is not implicated in the control of the emotional pain component [47]. Curiously, at 42 days after intraarticular injection, the increased activity in these pain- and emotional-behaviors-associated areas and the changes in the DNIC seem to rise in parallel with the presence of anxiety- and depressive-like behaviors, as suggested by the increased number of buried marbles and time spent in the open arms or in immobility. On the contrary, at 28 days we have found that monoarthritic rats exhibited only anxiety-like behaviors.

The development of anxio-depressive-like behaviors in chronic pain animal models has been reported in several studies [9,10,12,13,48]. Previous studies from our group have shown that anxiety-like behaviors in the CFA monoarthritis model are already present at 28 days [9,10,12]. Even though our group has also detected depressive-like behaviors at that time point [9], the observation of this emotional behavioral change at this exact period of progression of chronic pain conditions has not always been consistent between studies [13,48,49]. Moreover, the consensus in the literature is that in most cases, in chronic pain, these affective-related behaviors arise not simultaneously but in a time-dependent manner, i.e., the onset of anxiety- or depressive-like behaviors may happen weeks apart [13,50]. In the present study, we have shown that anxiety-like behaviors are already present at 28 days of monoarthritis and remain at 42 days, while depressive-like behaviors are only exhibited at day 42. Thus, our data seems to reinforce the idea that the time points for the onset of these two emotional comorbidities are, indeed, apart in time, at least in the monoarthritis CFA model. Extending the duration of the experimental study to six weeks of disease progression, as in the present study, allowed the detection of anxiety-like behaviors concomitantly with depressive-like behaviors. The late development of these comorbid anxiodepressive behaviors in chronic monoarthritis may raise the question whether these affective behaviors may possibly be associated in any way with the neuroplastic changes that occur in the nociceptive system during the chronicity of pain, which may be enough to impact the circuits of the affective component of pain. Among the neuroplastic changes in the nociceptive system, those occurring in the noradrenergic system are the most likely to affect the functioning of these circuits [9,12]. Indeed, it is quite interesting that these emotional comorbidities seem to be rising in parallel with the onset of crucial changes in the descending noradrenergic modulatory system.

The depressive-like behaviors were found only at the 42-day time point of monoarthritis. This behavior was observed at a time when we simultaneously observed significant changes in the noradrenergic system (attenuation of DNIC and molecular changes) and also a considerable increase in the neuronal activity in the LC (increased pERK1/2s) and consequently in supraspinal areas associated with the affective component, to which the LC projects. Even though, these data seem to suggest that the development of depressive-like behaviors might be associated with all these changes, further studies will be needed to explore this hypothesis. Additionally, in a study by Detke et al. (1996), it is suggested that changes in the swimming and climbing behaviors are associated with alterations in the serotoninergic and noradrenergic systems, respectively [51]. Indeed, at 42 days in the FST, we have found that the monoarthritic rats exhibited changes in the time spent swimming, while no differences were found in the time spent climbing, in comparison to the control group. Thus, it is extremely likely that a serotonergic component is also associated with the onset of these behaviors in the rats with late stage chronic monoarthritis. At the present moment, our group has an ongoing study exploring this hypothesis.

In contrast to the depressive-like behaviors, the anxiety-like behaviors were observed on days 28 and 42 after the intraarticular injections. Although we have not quantified pERKs labelling at 28 days in the present study, previous studies from our group in the monoarthritis model showed that anxiety-like behaviors were also present at this time point and that inhibition of ERK1/2 activation in the LC prevented the development of these behaviors at the same time point [12]. These data suggested there is some sort of causative association between ERK1/2 activation in the LC (and probably in its cortical and subcortical projections to emotion-related regions) and the development of anxiety during chronic joint pain. Moreover, in our study, contrary to what has been found at 42 days after intraarticular injection, DNIC was not attenuated, and there were no significant changes in the spinal levels of noradrenaline nor in spinal DBH labeling at 28 days, while anxiety-like behaviors were already present and were accompanied by mechanical hyperalgesia and movement-induced allodynia. Thus, our present data further suggests that the development of anxiety-like behaviors may not be directly associated with a noradrenergic impairment. However, this association may not be completely excluded as further studies are needed to completely clarify this hypothesis. Other experiments are necessary to investigate the involvement of other neurotransmitter systems, such as the serotoninergic and γ-aminobutyric acidergic (GABAergic) [52,53,54,55].

Our results show that the DNIC are present during chronic inflammatory joint pain but are attenuated with the maintenance of sustained noxious stimulation during prolonged periods of disease. This DNIC attenuation is accompanied by the recruitment of the noradrenergic system, which starts to be subsided at the same stage of the disease, as inferred by the changes in key molecular components of this neurotransmitter system. This may be somehow associated with a continuous activation of the LC, as a response to the increased demand of noradrenaline. Moreover, pain-induced anxiety- and depressive-like behaviors also are present at the same prolonged time point of chronic joint inflammation. Given the pattern of neuronal activation found in the LC and in the ACC and BLa, two brain areas implicated in emotion, and the different timeframe of manifestation of anxiety and depressive-like behaviors, it is likely that the development of depressive-like behaviors comorbidities may have a more direct implication of the noradrenergic system, while the anxiety-like behaviors may have the contribution of different mechanisms. More studies are needed to explore these hypotheses.

## 4. Materials and Methods

### 4.1. Subjects and Experimental Design

Adult male (*n* = 103) Wistar Han IGS rats (Charles River, Lyon, France) weighting 200–220 g, were housed two per cage, with ad libitum access to food and water, and they were maintained on a 12-h light–dark cycle at 22 °C and with 45% to 60% humidity. The adequate measures were taken to minimize pain and discomfort, and all experimental procedures were performed in accordance to the ethical guidelines for the study of experimental pain in animals and were carried out in accordance with the European Communities Council Directive of 22 September 2010 (2010/63/ EC). In addition, the work here presented was approved by the Faculty of Medicine of the University of Porto’s Ethical Committee for Animal Welfare and the Portuguese National Authority for Animal Health. All studies were carried out for 42 days after intraarticular injection of complete Freund’s adjuvant solution (CFA) or vehicle (see below).

### 4.2. Induction of Chronic Joint Inflammatory Pain Model: Monoarthritis

Monoarthritis was induced as previously described [9,56]. Under brief anesthesia with isoflurane (5% for induction and 3% for maintenance; Isoflo, Abbott Animal Health, Madrid, Spain), rats (*n* = 50) were injected in the left tibiotarsal joint with 50 μL of CFA solution (30 mg of desiccated *Mycobacterium butyricum* from Difco Laboratories, Detroit, MI, USA, diluted in the vehicle solution containing 3 mL paraffin oil, 2 mL saline, and 500 μL Tween-80). The control group (*n* = 51) was injected with CFA vehicle. Throughout the experimental period, the animals that showed signs of polyarthritis were immediately removed from the study.

### 4.3. Behavioral Evaluation

#### 4.3.1. Nociceptive Behavior and Inflammation: Assessment of Monoarthritis Evolution and Maintenance

To monitor the evolution and maintenance of monoarthritis, the nociceptive behavior and inflammatory signs were evaluated during the entire experimental period (42 days). Before starting the experiments, animals were habituated to the experimenter and testing conditions for at least seven days and for 5–10 min before each test on the testing days, until exploration activities ceased. Baseline values were obtained for all animals a day before the monoarthritis induction. After CFA or vehicle injection, tests were performed every week as described below.

#### 4.3.2. Assessment of the Inflammation Score

The inflammation signs were assessed on the ipsilateral paw on days 2, 4, and 7 during the first week for a stricter control of the monoarthritis evolution at early stages, and then once a week for the remaining 42 days. Joint inflammation and locomotor activity were assessed by using a previously described inflammation score, as follows: 0—no signs of paw inflammation or locomotor changes; 1—redness and swelling (minor changes) and no locomotor changes; 2—more intense redness and swelling and avoidance of passive movement; 3—more intense redness and swelling, avoidance of passive movement and reluctance to place weight over the affected limb; 4—severe inflammation and persistent flexion of the affected limb [57,58].

#### 4.3.3. Nociceptive Behavioral Testing

The rats were tested for movement-induced nociception (allodynia) and mechanical hyperalgesia, at several time points after CFA or vehicle injection, in the ipsilateral and contralateral paws. Movement-induced nociception was measured once a week, through the ankle bend test, as previously described [9,56]. For this purpose, the animals were lightly restrained by the experimenter and a sequence of five alternate flexions and extensions of the tibiotarsal joint was performed. The squeak and/or struggle reactions to each movement were recorded and scored according to the type of reaction and level of manipulation of the joint (moderate or maximal), in accordance with the following scale: 0—no response in any type of movement; 0.5—struggle to maximal flexion or extension; 1—squeak to maximal flexion or extension or struggle to moderate flexion or extension; 2—squeak to moderate flexion or extension. The final score equals the sum of all values attributed to each 10 reactions, and the maximum value is 20. The higher the ankle bend score, the higher the indication of allodynia. In addition, secondary mechanical hyperalgesia was measured by the Randall-Selitto test on days 7, 21, 35, and 42 [59]. For this test, increasing gradual pressure was applied to the paw using an analgesymeter (Ugo Basile, Milan, Italy), and the paw pressure value eliciting a paw withdrawal reaction was registered. Two measurements were taken for each paw within 5-min intervals. The test started at 30 g and had a cut-off of 250 g to avoid damage to the paw. Increased mechanical hyperalgesia is indicated by a lower paw pressure withdrawal value [9].

#### 4.3.4. Behavioral Analysis of Descending Modulation Controls (DNICs)

DNIC were evaluated on monoarthritic (*n* = 6) and control rats (*n* = 6) as described elsewhere [31]. All animals were habituated to the experimenter and testing conditions for at least one week before the intraarticular injections. A day before this procedure, the baselines for the inflammation score, ankle bend and Randall–Selitto tests were collected, as described above [9,59]. Behavioral analysis of DNIC was performed on days 7, 28, and 42 after CFA or CFA vehicle intraarticular injection. The animals were acclimated to the DNIC test once a day for two days before the testing day. For this purpose, the animals were restrained with a cloth and the tail was immersed in water at 36 °C for 2 min. During the experimental days, a pre-DNIC baseline was collected by using the Randall-Selitto test and, after 20 min, the animals were submitted to the DNIC inducting stimulus by immersing 7 cm of the tail (from the distal region onwards) in water at 47 °C for 2 min. Then, immediately after the stimulus, the Randall–Selitto test was performed again to measure the effect of DNIC on mechanical hyperalgesia. The final results are presented in terms of the amount of force (g) necessary for paw withdrawal before and after DNIC stimulation and also by the percentage of variation of the response (%) in relation to the pre-DNIC baseline, as follows: ∆ response = ((force for paw withdrawal post-DNIC (g)- force for paw withdrawal pre-DNIC (g))/ force for paw withdrawal pre-DNIC (g)) * 100. Furthermore, on the experimental days, the evaluation of the inflammation score and ankle bend test was also performed 30 min before DNIC evaluation, as an additional control of monoarthritis assessment.

#### 4.3.5. Anxiety- and Depressive-Like Behaviors

The development of pain induced anxiety- and depressive-like behavioral changes was assessed on days 28 and 42 after injection of CFA or CFA vehicle. The monoarthritic (*n* = 6 and *n*= 5 for days 28 and 42, respectively) and control groups (*n* = 6 and *n*= 5 for days 28 and 42, respectively) were acclimatized to the experimenter and the testing room for at least one week before the intraarticular injections and then once a week for the remaining experimental period. The marble burying (MB) and elevated zero maze (EZM) tests were used to evaluate the anxiety-like behaviors. For depressive-like behaviors, the forced swimming test (FST) was performed. To reduce the influence of the stress caused on the animals by the testing conditions, the tests were classified and organized from the least to the most stressful and thus were performed according to the following order: MB, EZM and FST. Additionally, all tests were separated by a 24-h interval.

#### 4.3.6. Anxiety-Like Behaviors and Locomotor Activity

Anxiety-like behaviors were evaluated by MB and EZM on days 28 and 42 after intraarticular injection. For the MB, the rats were acclimatized to the room for 30 min, as previously described [9,60]. Afterwards, they were individually placed in a plastic transparent cage (51 × 22 × 15 cm) illuminated with a 100 W light bulb and containing a 5-cm-deep bedding, where 20 marbles were arranged in four columns and five rows. The test lasted 30 min, after which the number of buried marbles was counted. The marbles were considered buried if they were at least two thirds covered with bedding. The higher the number of buried marbles by the monoarthritic group in comparison to the control group, the higher the indication of anxiety-like behavior.

On the EZM [9,61], animals were placed on a test arena, which consists of a black circular maze with 105 cm in diameter, elevated 65 cm above the floor. This platform is divided in four quadrants equal in length, but different in conformation with two opposing open quadrants and two closed. In order to avoid unwanted falls, the maze was protected all around its perimeter by a wall that was 1 cm high in the open quadrants and 27 cm on the closed ones. To begin the test, each animal was placed in the center of one of the closed quadrants and left to explore the maze for 5 min. The lighting conditions were the same for all trials, and each trial was recorded from above. The amount of time spent in the open arms (s) was analyzed, since more time spent in these areas indicates an anxiety-like behavior.

The EZM was also used to evaluate the animals’ locomotor activity by analysis of the distance travelled (cm) by the animals in the labyrinth during the EZM test. In the monoarthritis rat model, the diseased animals always present high inflammation scores in the ipsilateral paw, which affects the quality of the movement, thus causing in some animals the avoidance of passive movements with the affected limb [9]. Thus, the evaluation of locomotor performance through the parameter “distance travelled” was used to infer the validity of the obtained data, since significant differences between the groups regarding the travelled distance would invalidate data analysis. Spontaneous Motor Activity Recording and Tracking (SMART) software (Panlab S.L.U., Barcelona, Spain) was used to analyze the amount of time spent in the open arms (s) and the distance travelled (cm).

#### 4.3.7. Depressive-Like Behaviors

This type of behavior was evaluated by a modified version of the FST. The test was performed on days 28 and 42 after intraarticular injection, as described previously [9,13,62]. In brief, the animals were first submitted to a pre-test on the day before the FST. Each rat was placed for 15 min on a transparent cylindrical vessel filled with water, at 25 °C, up to 30 cm high. Then, on the test day (24 h later), the rats were individually placed in the same cylinders and under the same conditions, but for 5 min only. The testing session was recorded with a video camera. The analysis of the videos was performed by a blind observer and the following parameters were quantified: (1) latency to immobility (%; time spent since the beginning of the test until the moment when the animal first becomes immobile), (2) time spent immobile (%; floating with just enough movement to keep the head above the water), (3) time spent swimming (%; actively moving) and (4) time spent climbing (%; active forepaws movements, trying to escape from the cylinder) [13,63]. Depressive-like behavior (learned helplessness behavior) is established when the animal spends more time in immobility and has lower latency to immobility. On the day of this test, the FST conditions were validated using a positive control. Thus, a control group of naïve rats (*n* = 2) received an intraperitoneal injection of the antidepressant venlafaxine (20 mg/Kg; Sigma-Aldrich, St Louis, MO, USA) at 23.5, 5, and 1 h before the tests and underwent the FST, as described. Both animals showed no changes in the latency to immobility and spent more time in swimming and climbing activities and less time immobile, as expected (Table S4).

#### 4.3.8. Intrathecal Surgery

On day 35 after CFA or CFA vehicle intraarticular injection, an intrathecal sterile silicon catheter (internal diameter: 0.3 mm, outer diameter: 0.635 mm; Becton Dickinson & Co., Sparks, MD, USA) was inserted into the lumbar subarachnoid space of monoarthritic (*n* = 12) and control animals (*n* = 12), as described previously [64]. In brief, deep anesthesia was induced by a mixture of ketamine (75 mg/Kg; Imalgene™, Boehringer Ingelheim, Ingelheim am Rhein, Germany) and medetomidine (0.5 mg/Kg; Medetor™, Virbac, Sintra, Portugal) injected intraperitoneally, and the animals were submitted to a laminectomy at the level of thoracic spinal segments T8/T9. After piercing the meninges, a catheter was inserted through the subarachnoid space until the L4–L5 spinal cord segments were reached (length: 2.5 cm). The opposite end of the catheter was fixed with a suture to a layer of superficial muscles and was then externalized by being passed subcutaneously to the scapula region. The catheter was filled with 0.9% saline solution and sealed with quick glue gel to prevent cerebrospinal fluid leakage. All animals were individually housed and monitored daily for body weight and signs of motor deficit.

#### 4.3.9. Pharmacological Studies on Spinal a2-AR

**Figure 8:**
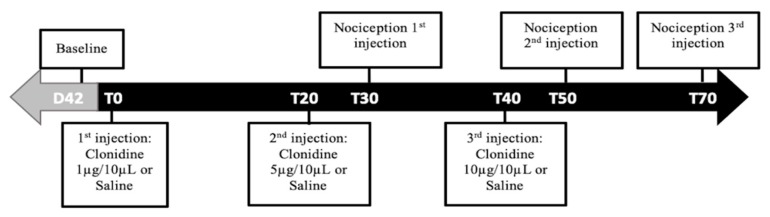
Pharmacological studies timeline. Three consecutive injections of saline or of increasing cumulative doses of clonidine hydrochloride (1, 5, and 10 μg) were administered through the catheter. All injections were separated by 20-min intervals. The Randall–Selitto test was used to evaluate the effects of each administration and was performed before the first intrathecal administration to collect a baseline value and 30 min after each injection.

These studies were performed to evaluate the functional activity of a2-AR at the L4–L5 spinal cord segments on day 42 of disease evolution. For this purpose, the animals were divided in four groups: (A) monoarthritic animals receiving intrathecal saline (monoarthritis + saline; *n* = 6); (B) monoarthritic animals receiving intrathecal clonidine, a centrally-acting a2-AR agonist (monoarthritic + clonidine; *n* = 6); (C) control animals receiving intrathecal saline (control + saline; *n* = 6); and (D) control animals receiving intrathecal clonidine (control + clonidine; *n* = 6). Then, on each animal, three consecutive injections of saline or of increasing cumulative doses of clonidine hydrochloride (1, 5 and 10 μg dissolved in saline solution [65]; Sigma-Aldridch, Saint Louis, MO, USA) were administered through the previously implanted catheter at a volume of 10 μL by using a 50 μL Hamilton microsyringe (Hamilton Inc., Reno, NV, USA). After each administration, 20 μL of saline were flushed to clean the catheter. All injections were administered over a period of 30 s and in intervals separated by 20 min. To evaluate the effects of each dose/injection, the Randall–Selitto test was performed on the ipsilateral and contralateral paws at the beginning of the experiment and 30 min after each injection (Figure 8). This timing was chosen based on clonidine’s pharmacokinetic properties. This drug evokes effects within 15 min after administration, lasting up to 60 min [66]. The most significant effects can be observed between 20–60 min [67]. For each dose, the results are expressed by the amount of force (g) eliciting a paw withdrawal observed before and after clonidine microinjection.

### 4.4. Neurochemical Studies

#### 4.4.1. Immunohistochemistry

The monoarthritic and control rats that were subjected to anxio-depressive-like behavioral evaluation were further used to evaluate the labelling of DBH (the biosynthetic limiting enzyme for noradrenaline) and a2-AR (subtype A (a2A-AR)), on the L4 and L5 spinal cord segments, to assess neurochemical changes on the spinal noradrenergic system. These molecular studies were first conducted at 42 days of monoarthritis (*n* = 5/group, for monoarthritic and control rats). When changes between groups were observed, the same quantification was performed at 28 days (*n* = 6, for monoarthritic and control rats).

On the brain, the labelling of pERK½ was also studied at 42 days of monoarthritis, at the LC, the basolateral amygdala (BLa), and the ACC, to assess the levels of neuronal activation on regions implicated in the affective component of pain perception. These proteins are markers of neuronal activity and are particularly active on supraspinal areas when painful or pain-induced emotional behaviors are present [29].

For this purpose, one day after all tests were concluded, the animals were terminally anesthetized intraperitoneally with sodium pentobarbital (0.5 mg/kg; Eutasil, MedVet, Bragança, Portugal) and perfused transcardially with Tyrode’s solution and 4% paraformaldehyde in phosphate-buffered saline (PBS) 0.1 M, pH 7.2. Then, the spinal cords (lumbar segments L1 to L6) and brains were dissected, submitted to a period of 4 h for post-fixation in the same fixative, and immersed on a 30% sucrose solution for 24 h. The tissue was then sliced with a freezing microtome (Leica CM 1325; Leica, Wetzlar, Germany) into sequential transverse sections of 30 µm (4 series) for the spinal cord and 40 µm (4 series) for the brain. Finally, the slices were stored in a solution of cryoprotector (phosphate buffer 0.1 M/Glycerol/ethylene glycol) at 20 °C until being used in free-floating immunohistochemical assays.

Light microscopy was used to detect spinal L4 and L5 DBH immunoreactive (IR) fibers and pERK½-IR cells on brain slices. For this, one series of spinal cord or brain sections was first washed in a 0.1 M PBS solution (three times; 10 min). After inhibition of the endogenous peroxidase activity with a solution of 1% hydrogen peroxide in PBS (15 min) and further washes in PBS (10 min) and PBST (10 min; 0.1 M PBS containing 0.3% Triton X-100), the sections were incubated for 2 h in a blocking solution containing 0.1M glycine and 10% normal horse serum (NHS) in PBST. Then, the sections were incubated with a specific primary antibody diluted in PBST with 2% NHS, as follows: (1) for spinal cord sections, one overnight at room temperature with a mouse primary antibody against DBH (1/5000; Millipore^®^, Burlington, MA, USA); (2) for brain sections, two overnights at 4 °C with a rabbit primary antibody against Phospho-p44/42MAPK (Thr202/Tyr204) (1/500; Cell Signaling Technology, Leiden, Netherlands). After further washes with PBST (three times; 10 min), the sections were incubated for 1 h, at room temperature, with a horse biotinylated anti-mouse secondary antibody (1/1000 in PBST with 2% NHS; Dako, Agilent Technologies, Glostrup, Denmark) for spinal cord or with a swine biotinylated anti-rabbit secondary antibody (1/1000; Molecular Probes, Eugene, OR, USA) for brain slices. Sections were washed again and incubated for 1 h in PBS-T containing the avidin-biotin complex (ABC) (1/200 in PBST with 2% NHS; Vector Laboratories, Burlingame, CA, USA). After washing in 0.05 M Tris hydrochloride (Tris-HCl), pH 7.6, the bound peroxidase was revealed using 0.0125% DAB (Sigma-Aldrich, Saint Louis, MO, USA) and 0.025% H_2_O_2_ in the same buffer. The sections were observed under a light microscope (Axioskop 40 model, Zeiss^®^, Hombrechtikon, Switzerland) coupled to a high-resolution digital camera (Leica EC3 model) and the LAS 4.6.0. software (Leica Microsystems^®^, Wetzlar, Germany).

For a2A-AR detection, immunofluorescence was used. One series of L4 and L5 spinal segments sections was washed in a 0.1 M PBS solution (three times; 10 min). After inhibition of non-specific background with a solution of 1% borohydride in PBS 0.1 M (15 min) and further washes in PBS and PBST, sections were incubated for 2 h in a blocking solution containing 0.1 M glycine and 10% normal goat serum (NGS) in PBST. Then, sections were incubated with a rabbit primary antibody against a2A-AR (1:500 in PBST with 2% NGS; Neuromics, Edina, MN, USA) for 2 h at room temperature and 2 overnights at 4 °C. Finally, after thorough washing, the sections were incubated with an Alexa 488 donkey anti-rabbit secondary antibody (1/1000; Molecular Probes, Eugene, OR, USA). Afterward, the sections were washed twice in PBST, then in PBS, and were rinsed in distilled water before being mounted on gelatin-coated slides in a low illuminated room. The slides were dried in the fridge for 1 h and then mounted with glycerol phosphate buffer to visualize under the fluorescent microscope. A negative control for this assay was performed simultaneously with the immunoreaction of all the samples. Thus, five spinal cord slices of naïve rats underwent the entire a2A-AR protocol with the exception of the incubation with the primary antibody, which was substituted by PBST with 2% NGS (Figure S1). Additionally, the specificity of the primary antibody has previously been shown by Chen et al. [68].

#### 4.4.2. Quantification of Immunolabelled Proteins

The quantification of the immunolabeled cells and fibers was performed with the assistance of the ImageJ^®^ software (US National Institutes of Health, Bethesda, MD, USA, free access). In order to assure an unbiased and standardized analysis, all quantifications were performed with a blind and randomized methodology and were performed by the same experimenter.

The quantification of DBH fibers and a2A-AR cells was done by densitometric analysis of the pixels, using a thresholding method [16,40]. Briefly, photomicrographs were taken from the ipsilateral and contralateral sides of the spinal dorsal horn of each animal. A total of five non-contiguous random sections from L4 and L5 (five sections per segment) were acquired under the same exposure and lighting settings. The densitometric analysis was performed on the total dorsal laminae I–VI. This quantification of the a2A-AR immunolabelling in the entire spinal dorsal horn was chosen to allow comparison with data obtained with the quantification of the western blot bands for the same receptors. Since the a2A-AR expression in the dorsal horn is mostly distributed on laminae I and II, we additionally performed the densitometric analysis of a2A-AR immunolabelling on these two superficial layers in order to exclude the effect of the dilution of the immunohistochemical signal and to further confirm our data. This data is presented in the Supplementary Figure 1 and Supplementary Table S3.

For DBH-labeled fibers, the thresholding analysis was performed by manually selecting a small random area of background and extracting the mean and standard deviation (SD) of the pixels’ intensity. Then, the images were converted to an eight-bit grayscale. The threshold level for DBH positive pixels was determined by setting a value of five SDs below the mean light background level, as follows: Threshold level (rounded to units) = mean background value – (5× SD). The regions of interest for this analysis, which comprised laminae I–VI of the dorsal horn, were delimited manually for each image. The percentage of DBH positive pixels inside the area of interest was automatically calculated by the ImageJ software. The quantification of a2-AR was performed using the same thresholding analysis as explained above, but with alterations specific for fluorescence. Briefly, the mean and SD values of background staining were obtained. Images in the 16-bit grayscale format were thresholded. The threshold level for a2-AR positive pixels was determined by setting a value of five SDs above the mean dark background level on the threshold tool, as follows: threshold level (rounded to units) = mean background value + (5× SD). The percentage of a2-AR positive pixels inside the area of interest was automatically calculated.

pERK1/2 immunoreactivity was analyzed at the LC, the ACC, the BLa, and the medial amygdala (Me). The Me, which is unrelated with pain-induced emotional behavior [47], was used as an internal control. The localization and delimitation of these areas was done in brain sections by using the Paxinos and Watson Rat Brain Atlas [69]. The rostrocaudal coordinates with respect to bregma were –9.16 mm to –10.52 mm for the LC, +3.70 mm to –1.40 mm for the ACC, –1.80 mm to –3.30 mm for the BLa and –1.30 mm to –2.80 mm for the Me. At the BLa, Me, and ACC [70], the quantification was done by counting the number of cell bodies with a brownish labelling for DAB, and the results were expressed in mean and SEM of the number of immunoreactive (IR) cells per section. The analysis of the rostrocaudal extension of the LC was performed using the same densitometric analysis as described above for DBH-IR cells.

### 4.5. Western Blotting

Western blotting analysis was used to quantify the levels of a2A-AR at the spinal cord. Intraarticular injections of CFA or CFA vehicle were performed to induce monoarthritis (*n* = 6) and control animals (*n* = 6), respectively, and the inflammation and the ankle bend scores were evaluated at baseline and weekly for 42 days to monitor the symptomatology. On the last day of the experimental period, the animals were sacrificed by decapitation, under deep anesthesia with isoflurane (5% for induction; Isoflo, Abbott Animal Health, Madrid, Spain), and the dorsal horns of the ipsilateral and contralateral sides of spinal cord L4 to L5 segments were freshly collected. The samples were immediately stored at –80 °C. For the western blotting assays, the samples were homogenized in lysis buffer composed by Tris-buffer saline with Tween 20 (TBST buffer: 20 mM Tris HCl pH 7.4; 150 mM NaCl; 0.1% Triton X-100) containing phosphatase inhibitor cocktail 2 (sodium orthovanadate, sodium molybdate, sodium tartrate and imidazole) and 3 (cantharidin, (-)-ρ-bromolevamisole oxalate and calyculin A; Sigma-Aldrich, Saint Louis, MO, USA) and protease inhibitor ([4-(2-aminoethyl) benzenesulfonyl fluoride hydrochloride], aprotinin, bestatin hydrochloride, -[*n*-(trans-epoxysuccinyl)-L-leucine 4-guanidinobutylamide], leupeptin, hemisulfate salt and pepstatin A; Sigma-Aldrich, USA), by using a MagNA Lyser^®^ (Roche, Switzerland). The total protein concentration was quantified by the Bradford method using Bovine Serum Albumin (BSA) protein as a standard. A total of 25 µg of protein was denatured at 60 °C for 10 min and centrifuged at 14800 rpm for 2 min in the 1x GLB (1.875 M Tris pH8.8; 15% glicerol; 6% SDS; 0.1%–0.05% bromophenol blue) containing 100 mM dithiothreitol (DTT). The samples were electrophoresed on 12% SDS-PAGE at 200 V and 32 mA. A pre-stained molecular weight marker (NZY Colour Protein Marker II^®^, NZYTech, Lisbon, Portugal) was simultaneously loaded to monitor electrophoresis and identify molecular weights. The proteins were then electroblotted onto nitrocelulose membranes by Trans-Blot^®^ TurboTM (BioRad, Hercules, CA, USA). After several washes with TBST, the membrane was blocked with non-fat milk (5% milk powder diluted in TBST buffer) for 1 h at room temperature and then incubated with the primary antibody rabbit anti-a2A-AR (1:1000, Neuromics, Edina, MN, USA) diluted in TBST containing 5% of non-fat milk, for 24 h at 4 °C. Membranes were then washed and incubated in anti-rabbit secondary antibodies conjugated to horseradish peroxidase (HRP, 1:10000, Jackson Immunoresearch Europe, Cambridge, UK) in TBST with 5% of non-fat milk for 1 h. After washing with TBST, the membranes were incubated with Clarity Western ECL Substrate (Bio-Rad, Hercules, CA, USA), a chemiluminescence reagent, for 5 min, and the immunoreative bands were detected by the Chemidoc system (Bio-Rad, Hercules, CA, USA). Semi-quantification of bands was performed using Image Lab software (Bio-Rad, Hercules, CA, USA) and was expressed in arbitrary units. Alpha-tubulin was used as a loading protein internal control, with the membranes being incubated with mouse anti-alpha-tubulin (1:10000, Abcam, Cambridge, UK) followed by incubation in anti-mouse secondary antibody conjugated to HRP (1:10000, Jackson Immunoresearch Europe, Cambridge, UK). Detection and quantification of alpha-tubulin immunoreactive bands were performed. The results of the quantification of a2A-AR expression were presented as normalized against alpha-tubulin.

### 4.6. High-Performance Liquid Chromatography (HPLC)

HPLC analysis was executed to quantify the levels of noradrenaline at the spinal cord at 28 and 42 days of monoarthritis. Thus, we performed intraarticular injections of CFA to induce monoarthritis (*n* = 6 and *n* = 9, for 28 and 42 days, respectively) or of CFA vehicle in control animals (*n* = 6 and *n* = 10, for 28 and 42 days, respectively). The evolution of the disease was monitored at baseline and weekly, through evaluation of the inflammation score and ankle bend test. At the end of each experimental period, all rats were sacrificed under deep isoflurane anesthesia (5% for induction; Isoflo, Abbott Animal Health, Madrid, Spain), and the ipsilateral and contralateral sides of the L4 and L5 spinal cord dorsal horns were freshly collected. The samples were placed in 0.2 M perchloric acid overnight at 4 °C, and then quantification of noradrenaline was performed by HPLC with electrochemical detection, as described previously [71]. For this, the aliquots of perchloric acid in which the samples had been maintained were placed in 5 mL conical-based glass vials with 50 mg alumina and the pH was adjusted to 8.6 with a Tris buffer. Then, the samples were mechanically shaken for 15 min and centrifuged at 2700 rpm for 2 min at 4 °C. After discarding the supernatant, the adsorbed noradrenaline was eluted with 200 μL of 0.2 M perchloric acid using Costar Spin-X microfilter tubes (Sigma-Aldrich, Saint Louis, MO, USA). Finally, 50 μL of the eluted solution were injected into the HPLC system, using as internal standard the 3,4-dihydroxybenzylamine. The HPLC system used for this assay has a Gilson model 302 pump linked to a Gilson model 802 C manometric module and a stainless-steel 5 μm ODS column (Biophase, Bioanalytical Systems, New Laffayette, IN, USA) with 25 cm in length. The automatic sample injector (Gilson model 231) is connected to a Gilson dilutor (model 401). For sample analysis, the mobile phase (degassed solution of citric acid (0.1 mM), sodium octylsulphate (0.5 mM), sodium acetate (0.1 M), EDTA (0.17 mM), dibutylamine (1 mM), and methanol (8% *v*/*v*), pH 3.5 with perchloric acid (2.0 M), was pumped at a rate of 1.0 mL/min. The detection of noradrenaline was achieved through a glass carbon electrode (Ag/AgCl reference electrode) and the Gilson model 141 amperometric detector, operated at 0.75 V. The lower limit of noradrenaline detection ranged from 350 fmol to 1000 fmol, and all standards were obtained from Sigma (St. Louis, MO, USA). The levels of noradrenaline were expressed in relation to the wet mass of spinal cord tissue.

### 4.7. Statistical Analysis:

All data were screened for normality assumptions using the Shapiro–Wilk test. Parametric tests were used whenever normality was achieved.

Two-way repeated measures ANOVA was used for time course analysis of the effects of monoarthritis in the nociceptive behavioral assays and also for the analysis of the effects of clonidine. The magnitude of DNIC on days 7, 28, and 42, as well as the anxio-depressive-like behaviors at 28 and 42 days, were compared by one-way ANOVA. Two-way and one-way ANOVA analysis were followed by the Tukey’s post hoc test for multiple comparisons, when appropriate.

For analysis of the labelling of a2A-AR (western blot and immunohistochemical assays), DBH, pERKs, and noradrenaline levels, the data collected at the ipsilateral and contralateral sides were first individually compared by the paired t-test, which yielded no statistically significant differences. Therefore, the values for both sides were then pooled and statistically compared.

For the DBH labelling and noradrenaline concentration on days 28 and 42, the data were compared by one-way ANOVA followed by the Tukey’s multiple comparisons post hoc test, when appropriate. For analysis of the a2-AR (western blot and immunohistochemical assays) and pERKs labelling, the unpaired t-test was used, except for western blot data which did not reach normality and was compared by the non-parametric Mann–Whitney test. The statistical analyses were performed using GraphPad Prism 7 software (GraphPad Software, Inc., La Jolla, CA, USA), and all data are presented as means ± standard error of the mean (SEM). The level of significance was set at a *p* < 0.05.

## Supplementary Materials

Supplementary materials can be found at https://www.mdpi.com/1422-0067/21/8/2973/s1.

## Abbreviations

a2-AR	α2-adrenergic receptors
ACC	Anterior cingulate cortex
BLa	Basolateral amygdala
CFA	Complete Freund’s adjuvant solution
DBH	Dopamine beta-hydroxylase
DNIC	Diffuse noxious inhibitory controls
ERK1/2	Extracellular signal-regulated protein kinases 1 and 2
EZM	Elevated zero maze test
FST	Forced swimming test
GABA	γ-aminobutyric acid-ergic
HPLC	High performance liquid chromatography
IR	Immunoreactive
MAO	Monoamino oxidase
MB	Marble burying test
Me	Medial amygdala
NET	Noradrenaline transporter
NRI	Noradrenaline reuptake inhibitors
pERK½	Phosphorylated extracellular signal-regulated protein kinases 1 and 2
