# Supplementary material for: Attenuation of the Diffuse Noxious Inhibitory Controls in Chronic Joint Inflammatory Pain Is Accompanied by Anxiodepressive-Like Behaviors and Impairment of the Descending Noradrenergic Modulation
Source: Int J Mol Sci. 2020 Apr 23;21(8):2973. doi: 10.3390/ijms21082973 (PMC7215719; doi:10.3390/ijms21082973)
Supplement: Supplementary file 1 [file ijms-21-02973-s001.pdf]

Supplementary

Supplementary Table S1. Statistical analyses data.

			Ipsilateral paw		Contralateral paw	
			F (DFn, DFd)	*P value*	*F (DFn, DFd)*	*p value*
**Two-way ANOVA with repeated-measures test**	**Behavioral tests**	*Inflammation score*	F (8, 80) = 98.13	*p* < 0.0001	N.A.	N.A.
		Ankle-bend	F (6, 60) = 102.3	*p* < 0.0001	N.A.	N.A.
		Randall Sellito	F (4, 40) = 10.64	*p* < 0.0001	F (4, 40) = 0.8809	*p* = 0.4840
	**DNIC experiments**	*Day 7*	F (2, 20) = 10.01	*p* = 0.0010	F (2, 20) = 0.1273	*p* = 0.8812
		Day 28	F (2, 20) = 5.336	*p* = 0.0139	F (2, 20) = 0.1686	*p* = 0.8460
		Day 42	F (2, 20) = 3.521	*p* = 0.0490	F (2, 20) = 0.5904	*p* = 0.5635
	**Pharmacologic experiments with clonidine**		F (3, 30) = 7.831	*p* = 0.0005	F (3, 30) = 1.490	*p* = 0.2371

For all experiments: values expressed in Mean ± SEM. Six animals per group. Two-way ANOVA with repeated-measures test for comparisons between the monoarthritic and control group. DNIC = diffuse noxious inhibitory control.

Supplementary Table S2. Statistical analyses data for the saline groups of the pharmacologic assays.

			Withrawal threshold (g)		BL versus t0		BL versus t30	
			Mean ± SEM		*t ratio*	*p value*	*t ratio*	*p value*
**Unpaired Student -t test**	**Baseline before the first clonidine administration (BL)**	*Monoarthritis ipsilateral (a)*	100.4 ± 7.6					
		Monoarthritis contralateral (b)	182.1 ± 15.8					
		Control ipsilateral (c)	185.8 ± 8.1					
		Control contralateral (d)	220.8 ± 8.7					
	**Saline**	**Time of saline administration**	**Before first injection (t0)**	**After first injection (t30)**				
		Monoarthritis ipsilateral (e)	105.8 ± 8.0	105.8 ± 11.0	0.4860	0.6374 (a/e)	0.3715	0.7180 (a/e)
		Monoarthritis contralateral (f)	189.6 ± 14.7	183.8 ± 11.2	0.3474	0.7355 (b/f)	0.0860	0.9332 (b/f)
		Control ipsilateral (g)	174.0 ± 32.8	197.1 ± 15.1	0.3492	0.7341 (c/g)	0.6559	0.5267 (c/g)
		Control contralateral (h)	228.3 ± 8.6	211.3 ± 13.6	0.6147	0.5525 (d/h)	0.5927	0.5665 (d/h)

Six animals per group. Unpaired Student-t test for comparison between groups. BL = baseline before the first clonidine administration (g); t0 = withdrawal threshold before the first saline injection (g); t30 = withdrawal threshold after the first saline injection (at 30 min) (g).

Supplementary Table S3. Statistical analyses data.

				F or t ratio	*p* value
**One-way ANOVA ; Student-t test; Non-parametric test**	**DNIC (d7 vs. D28 vs. D42)**			F (2, 13) = 8.469	*p* = 0.0044
	**Anxiety-like behavior (Monoarthritis vs. Control)**	*MB test*	*28D* / *42D*	F (3, 18) = 5.954	*p* = 0.0053
		EZM test	*28D* / *42D*	F (3, 18) = 10.52	*p*=0.0003
		EZM distance	*28D* / *42D*	F (3, 18) = 0.3436	*p* = 0.0003
	**Depressive-like behavior (Monoarthritis vs. Control)**	*FST immobility*	*28D* / *42D*	F (3, 18) = 18.75	*p*<0.0001
		FST swimming	*28D* / *42D*	F (3, 18) = 5.295	*p* = 0.0086
		FST climbing	*28D* / *42D*	F (3, 18) = 0.9808	*p* = 0.4238
		FST latency to immobility	*28D* / *42D*	F (3, 18) = 3.560	*p* = 0.0351
	**a2-AR (Monoarthritis vs. Control)**	*Immunolabelling L4 (Dorsal horn)*		t ratio = 1.366	*p* = 0.2092
		Immunolabelling L5 (Dorsal horn)		t ratio = 0.2586	*p* = 0.8025
		Immunolabelling L4 (Laminae I-II)		t ratio = 0.4781	*p* = 0.6454
		Immunolabelling L5 (Laminae I-II)		t ratio = 0.8915	*p* = 0.3987
		Western blot L4-L5		Mann-Whitney U = 8	*p* = 0.5000
	**Noradrenaline (Monoarthritis vs. Control)**		*28D* / *42D*	F (3, 27) = 3.468	*p* = 0.0299
	**DBH (Monoarthritis vs. Control)**	*Immunolabelling L4*	*28D* / *42D*	F (3, 18) = 3.959	*p* = 0.0249
		Immunolabelling L5	*28D* / *42D*	F (3, 18) = 2.930	*p* = 0.0417
	**pERK1/2 (Monoarthritis vs. Control)**	*LC*		t ratio = 4.345	*p* = 0.0025
		ACC		t ratio = 4.005	*p* = 0.0039
		BLa		t ratio = 4.571	*p* = 0.0018
		Me		t ratio = 2.099	*p* = 0.0691

DNIC experiments: Six rats per group. One-way ANOVA test for comparisons between days 7, 28 and 42. Anxiety-like behavior: six and five animals per group at 28 and 42 days, respectively. One-way ANOVA test for comparisons between groups in MB and EZM. Depressive-like behavior: six and five animals per group on days 28 and 42 after intraarticular injection, respectively. One-way ANOVA test for comparisons between groups in FST. a2-AR immunolabelling: five animals per group; Unpaired t-test for comparisons between groups in the densitometric analysis. a2-AR western blot: four animals per group; Mann–Whitney non-parametric test for comparisons between groups. Noradrenaline quantification: monoarthritic animals—six and nine animals per group on days 28 and 42, respectively. Control group: six and 10 animals per group on days 28 and 42, respectively. One-way ANOVA test for comparisons between groups. DBH immunolabelling: six and five animals per group at 28 and 42 days, respectively. One-way ANOVA test for comparisons between groups. D=Day; DNIC = Diffuse noxious inhibitory control; a2-AR = alpha2-adrenergic receptor; DBH = dopamine beta-hydroxylase; MB = marble burying test; EZM = elevated zero maze test; FST = forced swimming test.

Supplementary Table S4. Validation of the FST conditions.

	Venlafaxin group	
**FST parameters**	**Rat 1**	**Rat 2**
Immobility (s)	84	110
Swimming (s)	145	125
Climbing (s)	71	65
Latency to immobility (s)	44	72

Data concerning the venlafaxine group used as a positive control for the FST conditions. Two naïve rats received an intraperitoneal injection of the antidepressant venlafaxine (20 mg/Kg) and were subjected to the FST. Both animals showed no changes in the latency to immobility and spent more time in swimming and climbing activities and less time immobile, as expected. FST = Forced swimming test.

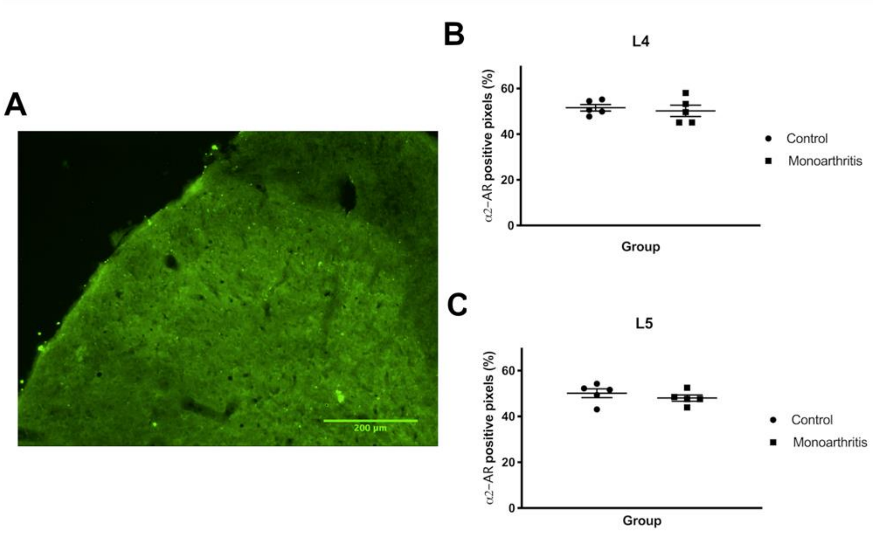

Supplementary Figure 1. Details regarding the immunohistochemical reaction for a2A-AR and the densitometric quantification of these spinal receptors. (A) Negative control performed simultaneously with the immunoreactions for a2A-AR in spinal cord slices of monoarthritic and control rats. In this control, the rabbit primary antibody against a2A-AR (1:500) from the company Neuromics (USA) was replaced with PBST with 2% NGS, while the rest of the protocol remained unaltered. (B-C) Densitometric analysis of the immunofluorescence labelling for a2A-AR in the spinal dorsal horn laminae I-II of L4 (B) and L5 (C) segments in control and monoarthritic rats. No significant changes were detected in the percentage of a2A-AR positive pixels between the two groups in both spinal segments. Values expressed in Mean ± SEM. Unpaired t-test for comparisons between the monoarthritic and control group in the immunofluorescence; five animals per group.
